# Predictive factors associated with prolonged survival in patients with advanced non-small-cell lung cancer (NSCLC) treated with gefitinib

**DOI:** 10.1038/sj.bjc.6603710

**Published:** 2007-03-27

**Authors:** M Satouchi, S Negoro, Y Funada, Y Urata, T Shimada, S Yoshimura, Y Kotani, T Sakuma, H Watanabe, S Adachi, Y Takada, Y Yatabe, T Mitsudomi

**Affiliations:** 1 Hyogo Medical Center for Adults, Respiratory Medicine, Akashi, Japan; 2 Hyogo Medical Center for Adults, Pathology, Akashi, Japan; 3 Hyogo Medical Center for Adults, Radiology, Akashi, Japan; 4 Aichi Cancer Center Hospital, Pathology and Molecular Diagnosis, Nagoya, Japan; 5 Aichi Cancer Center Hospital, Thoracic Surgery, Nagoya, Japan

**Keywords:** epidermal growth factor receptor (EGFR) inhibitor, EGFR mutations, gefitinib, IRESSA, non-small-cell lung cancer, smoking

## Abstract

This study aimed to identify predictive factors associated with prognostic benefits of gefitinib. A total of 221 Japanese patients who received gefitinib (250 mg day^−1^) were examined retrospectively and potential predictive factors analysed. Overall response rate (ORR) was 24.4% and median survival time (MST) was 8.0 months. In a log-rank test, survival was significantly better in females, patients with adenocarcinoma, never-smokers, favourable performance status (PS) and patients with epidermal growth factor receptor (EGFR) mutation. The lower the smoking exposure (Brinkman Index (BI)=cigarettes per day × years smoked), the better the MST (BI 0: 14.5 months, BI <500: 9.5 months, BI 500 to <1000: 6.9 months, BI ⩾1000: 4.0 months). Positive-EGFR mutation status and PS 0–1 were independent predictors of favourable prognosis by multivariate analysis. Prognosis was significantly different according to EGFR mutation status (with the same smoking status), but not according to smoking status (with the same EGFR mutation status). EGFR mutation status is the most important independent predictor of survival benefit with gefitinib treatment. Although differences in prognosis were observed according to relative smoking status and smoking exposure, the results suggested that smoking is not a direct predictor of prognosis, yet is a surrogate marker of EGFR mutation status.

Gefitinib (IRESSA) is an orally active small-molecule compound that inhibits the epidermal growth factor receptor (EGFR) tyrosine kinase (TK) by competing with adenosine triphosphate (ATP) at the ATP-binding site. In two large Phase II trials (IDEAL: IRESSA Dose Evaluation in Advanced Lung cancer 1 and 2) gefitinib-induced tumour regression and provided symptom relief in previously treated patients with non-small-cell lung cancer (NSCLC) (Fukuoka *et al*, 2003; Kris *et al*, 2003). Although a placebo-controlled Phase III study (ISEL) in previously-treated patients with NSCLC has not shown a statistically significant improvement in survival associated with gefitinib, preplanned subgroup analysis suggested survival benefits in patients of Asian origin and never-smokers (Thatcher *et al*, 2005). Patient selection criteria were not incorporated in this comparative study, which most likely contributed to the absence of a positive survival benefit in the overall population. In fact, a Phase II study in which gefitinib was used as first-line therapy for NSCLC in a subgroup of never-smokers with adenocarcinoma reported favourable outcomes, with an overall response rate (ORR) of 61% (Lee *et al*, 2005).

In 2004, mutations in the *EGFR* gene conferring increased sensitivity to gefitinib were reported (Lynch *et al*, 2004; Paez *et al*, 2004). Recently, very favourable outcomes (response rate (RR) 75%) in a Phase II study of gefitinib as first-line therapy for patients with NSCLC with *EGFR* gene mutations has been reported (Inoue *et al*, 2006).

Therefore, it is important to conduct patient selection before using gefitinib and, in particular, it is vital to identify the predictive factors that may contribute to survival. To aid future selection of patient groups for gefitinib treatment we conducted a retrospective analysis of patients who had been treated with gefitinib, assessing the relationship between clinical characteristics, the EGFR mutation status, antitumour activity and patient survival.

## PATIENTS AND METHODS

### Patients

A total of 221 patients who had been initiated on gefitinib monotherapy (250 mg day^−1^) during a 3-year span from July 2002 (gefitinib was launched in Japan) to June 2005 at the Hyogo Medical Center for Adults in Japan were retrospectively examined.

### Clinical assessments

Clinical parameters studied were gender, age, smoking history (Brinkman Index (BI)=number of cigarettes per day × number of years smoked), Eastern Cooperative Oncology Group performance status (PS) and previous lines of chemotherapy.

Assessment of tumour regression was conducted according to the response evaluation criteria in solid tumours (RECIST) guideline. The National Cancer Institute Common Toxicity Criteria, version 3.0, was used to evaluate toxicity.

### *EGFR* gene analysis

EGFR gene mutation detection was performed on samples from 106 patients: surgical specimens were obtained from 34 patients and a transbronchial lung biopsy (TBLB) was performed on 72 patients. EGFR mutation analysis was successfully performed in 91 of the 106 samples. EGFR mutation was analysed at Aichi Cancer Center Hospital in Japan. A cycleave PCR technique for codon 858 of *EGFR* gene was used on a SmartCycler system (SC-100, Cepheid, Sunnyvale, CA, USA). Deletion in exon 19 of the *EGFR* gene was detected with fragment analysis using an ABI PRISM 310 genetic analyzer (Applied Biosystems, Foster City, CA, USA) (Yatabe *et al*, 2006). Many of the cases began treatment on gefitinib before it had been reported that EGFR mutation detection was important when treating with the drug. Moreover, many of those cases had already died before our plans to undergo EGFR mutation detection, effectively preventing us from obtaining informed consent in this regard. Accordingly, our Institutional Review Board approved our study plan, provided that samples would be processed anonymously, that samples would be analysed only for somatic mutations and not germline mutations, and that the presence of the study be publicly disclosed, strictly according to the ‘Ethical Guidelines for Human Genome Research’ published by The Ministry of Education, Culture, Sports, Science and Technology, The Ministry of Health, Labour and Welfare and The Ministry of Economy, Trade and Industry, Japan. (http://www.mext.go.jp/a_menu/shinkou/seimei/genome/04122801.htm).

### Statistical analysis

OS analysis was conducted on all 221 and 91 patients in which EGFR mutation analysis could be successfully performed.

The differences in responders (complete response; CR+partial response; PR) by each factor (gender, PS, histology, prior chemotherapy, smoking status and mutation status) were examined with the Fisher's exact test. The difference in mutation rate among groups categorised by smoking exposure was examined with *χ*^2^ test.

An OS curve was plotted using the Kaplan–Meier method and survival curve comparisons were conducted with the log-rank test. Univariate analysis and multivariate analysis of the impact of the factors, including gender (male vs female), smoking history (ever-smokers vs never-smokers), histology (adenocarcinoma vs others), PS (PS 0–1 vs 2–4) and EGFR mutation (positive vs negative) were conducted using the Cox regression model. All analysis was determined to be statistically significant where the *P*-value was <0.05.

Analyses were conducted using the SPSS 11.0.1.

## RESULTS

### Patient characteristics

The clinical characteristics of the patients are shown in Table 1. The majority of patients (89%) had adenocarcinoma histology. One hundred and thirty-one patients (59%) were ever-smokers.

### EGFR mutation analysis and clinical response

TBLB or surgical samples were available from 106 patients for EGFR-mutation detection, but actual analysis was only possible for 103 patients because tumour cells were not found in three post-treatment specimens.

DNA could not be amplified in 12 cases. Analysis of the remaining 91 samples showed EGFR mutations in 28 patients (30.8%) and wild type in 63 patients (69.2%). EGFR mutation rate was high in females, never-smokers and patients with adenocarcinoma. Among ever-smokers, EGFR mutation rate was higher in patients with BI<500 and BI 500 to 1000 than BI >1000 (Table 2). Of the 28 EGFR mutations, 19 (67.9%) were exon 19 in-frame deletions and nine (32.1%) were exon 21-point mutations (L858R). Seven (36.8%) of the exon 19 deletions and four (44.4%) of the L858R cases were smokers. Significantly high mutation rates were observed in females and never-smokers.

In the overall population, RR was 24.4% (95% confidence interval (CI) 18.0–30.6%) (Table 1). Response rate was significantly higher among females, patients with adenocarcinoma histology, never-smokers and patients with the EGFR mutation. Disease control rate (DCR: CR+PR+stable disease; SD) was 51.1% (95% CI 44.3–57.9%) and among those with EGFR mutation, 100%.

### Survival analyses

Median survival time (MST) in the overall population was 8 months, with 34.8% surviving 1 year. MST among patients showing PR was significantly longer than that in the SD cases (*P*=0.003) and MST of the SD cases was also shown to be significantly longer than that of the PD cases (*P*<0.0001) (Table 1).

**Figure 1:**
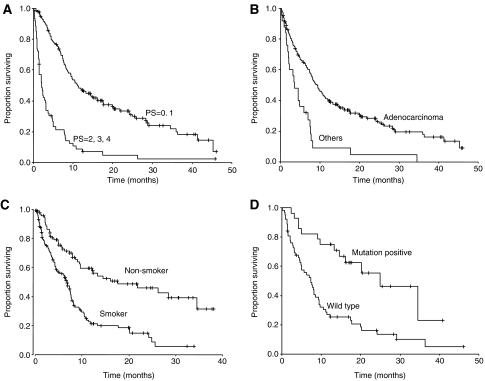
Kaplan–Meier plots of survival for patients receiving gefitinib treatment classified according to (**A**) PS, (**B**) histology, (**C**) smoking status and (**D**) *EGFR* gene mutation status.

Kaplan–Meier curves indicated significantly longer survival in patients with favourable PS (*P*<0.0001), in patients with adenocarcinoma histology (*P*<0.0001), in never-smokers (*P*<0.0001), and in patients with EGFR mutations (*P*<0.0001) (Table 1, Figure 1). L858R patients tend to survive longer than those with deletions at exon 19 (*P*=0.0539). Multivariate analysis was conducted to identify factors contributing to survival. When all patients were analysed considering of the clinical characteristics (gender, adenocarcinoma histology, smoking status and PS), adenocarcinoma, never-smoker status and PS 0–1 were found to be prognostic factors of survival (Table 3a). However, analysis (including that on EGFR mutation status and clinical characteristics) of the patients for whom EGFR mutation results were obtained showed PS 0–1 and *EGFR* gene mutation status to be the independent prognostic factors, and the relationship between smoking status and survival did not reach statistical significance (Table 3b).

**Figure 2:**
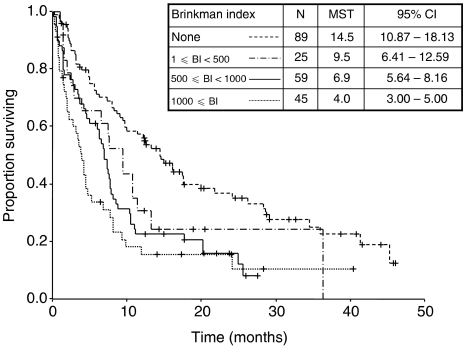
Survival stratified by smoking exposure (classified by BI).

**Figure 3:**
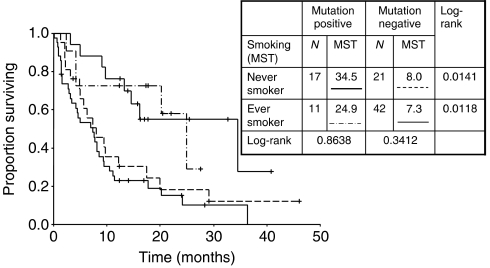
Survival stratified by smoking status and *EGFR* gene mutation status.

Further analysis of smoking exposure and survival indicated that the higher the exposure, the shorter the MST (Figure 2) . The presence of EGFR mutation was associated with significantly prolonged survival in both never-smokers (*P*=0.014) and ever-smokers (*P*=0.012). Furthermore, among EGFR mutation-positive patients, there was no statistically significant difference in median survival between never-smokers and ever-smokers (*P*=0.864), although patient numbers were small (Figure 3).

### Tolerability

Adverse events were observed in 165 out of 221 (75%) patients. Common adverse events were rash/dry skin (51%), diarrhoea (22%), liver dysfunctions (20% (2.3% were Grade 3)) and paronychia (14%). Sixteen (7%) of the patients developed interstitial lung disease (ILD) and three (1.4%) died. As three out of 14 (21%) patients with PS 3 developed ILD, patients with poorer PS were more likely to develop ILD. There were no differences in ILD incidence by gender, smoking history, age or histology. ILD was experienced by four out of 63 patients with wild type and two out of 28 patients with EGFR mutation (both with an exon 19 deletion).

## DISCUSSION

The data from this retrospective study suggest that in a practical setting

1. A favourable PS, adenocarcinoma histology, never-smoking and presence of an EGFR mutation are predictive of increased antitumour activity with gefitinib,
2. Although PR cases showed longer median survival than SD cases, SD cases also displayed significantly longer median survival than PD cases,
3. Although, in terms of clinical characteristics, PS 0–1, adenocarcinoma histology and never-smoking status are predictive factors of survival with gefitinib in the overall population, PS 0–1 and EGFR mutation status were identified as independent predictive factors in patients in which EGFR mutation status has been detected,
4. The relationships between smoking/EGFR mutation status and survival suggest that the latter is more related to prognosis. Conceivably, smoking has a strong confounding relationship with EGFR mutation status and smoking exposure can result in a different prognosis.

IDEAL 1 reported favourable antitumour activity in females and patients with adenocarcinoma histology (Fukuoka *et al*, 2003). Several subsequent retrospective studies have reported that female gender, adenocarcinoma histology, bronchioloalveolar subtype, never-smokers and patients with favourable PS are predictive factors of response (Miller *et al*, 2004; Kim *et al*, 2005; Lim *et al*, 2005; Ando *et al*, 2006). EGFR mutation has been reported as a predictor of efficacy of gefitinib and erlotinib (Lynch *et al*, 2004; Pao *et al*, 2004). There have been several reports of clinical factors associated with EGFR mutations, and per the univariate analysis, mutation frequency is high in patients of East Asian ethnicity, females, never-smokers and adenocarcinomas (Kosaka *et al*, 2004; Paez *et al*, 2004; Shigematsu *et al*, 2005; Tokumo *et al*, 2005). Moreover, multivariate analysis has shown that adenocarcinoma histology and never-smoker status are independent factors associated with EGFR mutation (Kosaka *et al*, 2004; Tokumo *et al*, 2005). Reports to date have shown that approximately 90% of EGFR mutations are centred around the L858R point mutation in exon 21 and deletions centred around codons 746–750 in exon 19 (Kosaka *et al*, 2004; Shigematsu *et al*, 2005; Sonobe *et al*, 2005; Tokumo *et al*, 2005). As association between these two types of EGFR mutations and the antitumour activity and prolonged survival with gefitinib has been reported (Han *et al*, 2005; Mitsudomi *et al*, 2005), we conducted analysis on these two types of mutations only. Our results were compatible with those from prospective Phase II studies conducted in patients with EGFR mutation (Inoue *et al*, 2006; Okamoto *et al*, 2006). Epidermal growth factor receptor mutation therefore appears to be a more specific criterion for gefitinib use than patient selection according to clinical characteristics.

The relatively high incidence of ILD (3.5–5.8%) in patients treated with gefitinib has been reported in Japan. It also revealed that male gender, ever-smokers, poor PS and the coincidence of interstitial pneumonia were predictive factors for the development of ILD (Yoshida, 2005; Ando *et al*, 2006). Although these predictive factors contrast with those for the presence of an EGFR mutation, two of the 28 patients with an EGFR mutation developed ILD in our study.

In our examination of prognostic factors, we analysed the relationship particularly between smoking status (ever-/never-smoker, smoking exposure) and two types of EGFR mutations, as well as the relationship between smoking status and EGFR mutation. Our findings indicated that patients with EGFR mutation had significantly longer MST in both ever- and never-smokers, and there was no significant difference in MST between ever- and never-smokers with the same mutation status. This led to the conclusion that the essential factor associated with survival is EGFR mutation status. Though better MST has been reported in L858R cases in a comparison of survival between exon 19 deletion and L858R missense (Shigematsu *et al*, 2005), recent reports have shown better survival in patients with exon 19 deletion (Jackman *et al*, 2006; Riely *et al*, 2006). Incidentally, we found MST to be better in L858R cases. As reported by Jackman *et al* (2006), RR was more favourable among the exon 19 deletion cases. Although this was conceivably due to factors including the ILD being experienced in two cases with exon 19 deletion and the impact of post-gefitinib treatment, the relatively small sample did not allow for any clarification in this respect.

Our data also show that the larger the smoking exposure, the shorter the survival.

There have been several reports of an inverse correlation between smoking exposure and EGFR mutation rate (Han *et al*, 2005; Pham *et al*, 2006; Sugio *et al*, 2006; Tam *et al*, 2006; Toyooka *et al*, 2006). In line with these studies, our data show that smoking status, unlike EGFR mutation status, is not an independent prognostic factor. Considered in combination with past reports on smoking exposure and EGFR mutation rates, the inverse correlation between smoking exposure and MST shown by our data might conceivably reflect that mutation rates differ according to smoking exposure. They also indicate that smoking status is a very powerful surrogate marker of EGFR mutation status, which is a prognostic factor for prolonged survival with gefitinib treatment. Our multivariate analysis in terms of clinical characteristics indicates that smoking status is a significant predictor. However, the multivariate analysis adding EGFR mutation status eliminates the significant difference with regard to smoking, demonstrating that EGFR mutation status and PS 0–1 are independent prognostic factors. This also suggests that ECOG PS and EGFR mutation status are factors that can be used to predict the intrinsic effect of gefitinib on patients as well as their prognosis, supporting the claim that smoking could be a surrogate marker of EGFR mutation status for prediction of survival benefit. Although RR in never-smokers and cases with EGFR mutation on erlotinib, which is also an EGFR-TKI, has been significantly favourable, there has only been marginal significant interaction between survival and smoking status (Clark *et al*, 2006). However, no significant differences have been reported in regard to EGFR mutation status, and detection of the EGFR mutation is considered unnecessary in treatment using erlotinib (Tsao *et al*, 2005; Clark *et al*, 2006). Our results showing that EGFR mutation and smoking status can function as predictors of survival benefit differ from reports on erlotinib. However, they concur with reports to date on gefitinib, presumably suggesting the necessity to select patients before using gefitinib. Further clinical studies are warranted to examine the survival benefits of gefitinib according to EGFR mutation status, that is, to make the EGFR mutation status an inclusion criterion. Considerations should be made in clinical practise to analyse actively EGFR mutations status where possible. However, it is often very difficult to obtain histological specimens of advanced and recurrent lung cancer for which gefitinib is indicated. In fact, in this study we were only able to obtain analytical results for EGFR mutation status for 91 out of 221 (42%) patients. Another problem is EGFR mutation analysis takes time, about 1–3 weeks, necessitating a wait-time before treatment. Therefore, when a certain clinical environment does not allow for, or complicates the detection of EGFR mutations, smoking exposure/smoking status could be a quick and inexpensive reference as a surrogate marker of EGFR mutation status. In future, it will be necessary to evaluate the survival benefits of gefitinib via a Phase III study in patients with these better predictive factors.

IRESSA is a trademark of the AstraZeneca group of companies.

## Figures and Tables

**Table 1:** Demographics and relationship between clinical variables and antitumor response/overall survival in patients treated with gefitinib

**Characteristic**	**No. of patients (%)**	**PR (n)**	**RR (%)**	**(95% CI)**	*P*-value*	**MST (months)**	**(95% CI)**	***P*-value**
All	221	54	24.4	(18.9–30.6)		8	(6.66–9.34)	
								
*Gender*								
Male	142 (64)	20	14.1	(8.8–20.9)	<0.001	6.8	(5.04–8.56)	0.036
Female	79 (36)	34	43	(31.9–54.7)		13.3	(8.84–17.76)	
								
*Age*								
65<	100	20	20	(12.7–29.2)	0.208	9	(6.41–11.59)	0.2852
<65	121	34	28.1	(20.3–37.0)		7.3	(5.88–8.72)	
								
*ECOG PS*								
0–1	160 (72)	44	27.5	(20.7–35.1)	0.114	11.1	(8.30–13.90)	<0.001
2–4	61 (28)	10	16.4	(8.2–28.1)		2.1	(1.26–2.94)	
								
*Histology*								
Adenocarcinoma	196 (89)	52	26.5	(20.5–33.3)	0.048	9.3	(7.66–10.94)	0.137
Others	25 (9)	2	8	(1.0–26.0)		3.6	(2.13–5.07)	
								
*Prior chemotherapy*								
Yes	188 (85)	45	24.6	(18.5–43.3)	1	8.1	(6.67–9.53)	1
No	33 (15)	9	25.7	(12.5–43.3)		8.4	(5.72–11.08)	
								
*Smoking history (*n*=220)*								
No	89 (40)	37	41.6	(31.2–52.5)	<0.001	14.5	(10.87–18.13)	<0.001
Yes	131 (15)	17	13	(7.7–20.0)		6.5	(4.36–8.64)	
BI 1<500	25 (11)	5	20	(6.8–40.7)		9.5	(6.41–12.59)	
BI 500 to <1000	59 (27)	9	15.3	(7.2–27.0)		6.9	(5.64–8.16)	
BI⩾1000	45 (21)	2	4.4	(0.5–15.1)		4	(3.00–5.00)	
*EGFR gene status (*n*=91)*								
Wild type	63 (69)	7	11.1	(4.6–21.6)	<0.001	7.4	(4.84–9.96)	<0.001
Mutation positive	28 (31)	20	71.4	(51.3–86.8)		24.9	(14.27–35.53)	
Exon 19 deletion	19 (21)	15	78.9	(54.4–93.9)		16.1	(6.22–25.98)	
Exon 21 (L858R)	9 (10)	5	55.6	(21.2–86.3)		>34.5	—	
								
*Tumor response (n*=*191)*								
PR	54	—	—			26.2	(15.76–36.64)	0.003
SD	59	—	—			11.9	(7.47–16.33)	<0.0001
PD	78	—	—			5.6	(3.20–8.00)	

Abbreviations: BI=Brinkman Index; BI=defined as number of cigarettes per day × number of years smoking; CI=confidence interval; EGFR=epidermal growth factor receptor; ECOG PS=Eastern Cooperative Oncology Group performance status; MST=median survival time; PD=progressive disease; PR=partial response; RR=response rate; SD=stable disease.^*^Fisher's exact test.

**Table 2:** Mutation rate by patient background

**Population**	***N***	**Mutation (%)**	**95%CI**	***P*-value^a^**
All samples	91	28 (30.8)		
Male	59	12 (20.3)	11.0–32.8	0.005
Female	32	16 (50.0)	31.9–68.1	
Never-smoker	38	17 (44.7)	28.6–61.7	0.014
Ever-smoker	53	11 (20.8)	10.8–34.1	
Adeno	81	27 (33.3)	23.2–44.7	0.166
Non-adeno	10	1 (10.0)	0.3–44.5	
				
*Brinkman Index*				
0	38	17 (44.7)	28.6–61.7	0.055^b^
1≪500	9	2 (22.2)	2.8–60.0	
500≪1000	25	7 (28.0)	12.1–49.4	
1000<	18	2 (11.1)	1.4–34.7	

a Fisher's exact test (two-sided).

b *χ*^2^-test (likelihood ratio).

**Table 3a:** COX Proportional Hazard Model for Survival Analysis in Overall Population (*N*=221)

**Variable**	**HR**	**95%CI**	***P*–value**
Never-smoker/Ever-smoker	0.413	0.294–0.582	<0.001
Adeno/Non-adeno	0.416	0.265–0.654	<0.001
PS 0, 1/2–4	0.205	0.145–0.291	<0.001

Stepwise method (include <0.05, exclude >0.2).

Tested variables; gender, smoking, histology, PS, excluded variable; gender.

**Table 3b:** COX proportional hazard model for survival analysis in patients in which EGFR mutation status has been detected (*n*=91)

**Variable**	**HR**	**95%CI**	***P*-value**
Adeno/Non-adeno	0.581	0.288–1.171	0.129
Never-smoker /ever-smoker	0.607	0.351–1.048	0.073
Mutation negative/positive	2.543	1.345–4.808	0.004
PS 0, 1/2–4	0.166	0.091–0.303	<0.001

Tested variables; gender, smoking, histology, PS, mutation excluded variable; gender.
